# Risk for Behçet’s disease gauged via high-density lipoprotein cholesterol: a nationwide population-based study in Korea

**DOI:** 10.1038/s41598-022-17096-0

**Published:** 2022-07-26

**Authors:** Yeong Ho Kim, Hyun Jee Kim, Jin Woo Park, Kyung Do Han, Yong Gyu Park, Young Bok Lee, Ji Hyun Lee

**Affiliations:** 1 Department of Dermatology, College of Medicine, Seoul St. Mary’s Hospital, The Catholic University of Korea, 222, Banpo-daero, Seocho-gu, Seoul, 06591 Republic of Korea; 2 Department of Dermatology, College of Medicine, Eunpyeong St. Mary’s Hospital, The Catholic University of Korea, Seoul, Republic of Korea; 3 Department of Statistics and Actuarial Science, Soongsil University, Seoul, Republic of Korea; 4 Department of Biostatistics, College of Medicine, The Catholic University of Korea, Seoul, Republic of Korea; 5 Department of Dermatology, College of Medicine, Uijeongbu St. Mary’s Hospital, The Catholic University of Korea, 271 Chunbo Street, 07345 Uijeongbu, Seoul, Republic of Korea

**Keywords:** Diseases, Medical research, Rheumatology

## Abstract

Behçet’s disease (BD) is a chronic inflammatory disease. Low levels of plasma high-density lipoprotein cholesterol (HDL-C) are associated with Crohn’s disease, another chronic inflammatory disease. However, the effects of low HDL-C levels on BD are unclear. We investigated the effects of HDL-C levels, and variability therein, on the risk for BD. We used the Korean National Health Insurance System database to identify 5,587,754 adults without a history of BD who underwent ≥ 3 medical examinations between 2010 and 2013. Mean HDL-C levels at each visit were used to calculate variability independent of the mean (VIM) and the coefficient of variation (CV). There were 676 new cases of BD (0.012%). The risk for BD was increased in participants with highly variable and low mean HDL-C levels. In a multivariate-adjusted model, the hazard ratios (95% confidence intervals) for BD incidence were 1.335 (1.058–1.684) in a high mean/high VIM group, 1.527 (1.211–1.925) in a low mean/low VIM group, and 2.096 (1.67–2.63) in a low mean/high VIM group compared to a high mean/low VIM group. Low mean HDL-C levels, and high variability therein, are independent risk factors for BD.

## Introduction

Behçet’s disease (BD) is a chronic inflammatory disease that causes oral and genital ulcers, uveitis, and skin lesions^1^. BD is a multisystem vasculitis that affects the mucosal lining, eyes, and joints as well as the nervous, cardiovascular, and gastrointestinal systems. The incidence and prevalence of BD are highest in areas near the ancient Silk Road, particularly Korea. The annual incidence rate of BD in Korea is 3976/100,000 population, much higher than in other countries^2,3^. Although the pathomechanism of BD is not clear, abnormalities of the immune system are probably involved^4^.

Lipid abnormalities increase the risk for atherosclerosis, and low levels of high-density lipoprotein cholesterol (HDL-C) in particular significantly increase the risk for cardiovascular disease^5,6^. Variability in cholesterol levels has been recognized as a risk factor for many diseases^7–10^. Low mean and highly variable HDL-C levels are associated with an increased risk for diabetes, myocardial infarction (MI), stroke, and mortality^7,11^.

The main pathological findings in BD are vasculitis and endothelial damage. Endothelial dysfunction leads to focal lipid deposition in the arterial intima layer^12,13^. In BD patients, the levels of atherosclerotic and inflammatory markers, such as tumor necrosis factor-α (TNF-α), lipoprotein-associated phospholipase A2 (Lp-PLA2), and homocysteine, are increased^14^. Recent studies have demonstrated low HDL-C levels in patients with Crohn’s disease, a chronic inflammatory disease^15^, but the relationship between HDL-C levels and BD is unclear. We investigated the effects of HDL-C levels (mean levels and variability therein) on the risk for BD in a national, population-based cohort of over 5 million people.

## Results

### Baseline characteristics of the study population

Table 1 summarizes the baseline characteristics of participants in groups based on HDL-C mean and CV. Participants were divided into four groups: high-mean/low-variability, high-mean/high-variability, low-mean/low-variability, and low-mean/high-variability groups. Compared to participants in the other groups, those in the low-mean/high-variability group had higher age, BMI, waist circumference, triglyceride levels, and prevalence of DM and hypertension as well as lower total cholesterol levels and income.

**Table 1:** Baseline characteristics of participants according to HDL-C levels (mean value and variability therein).

	High mean/low variability	High mean/high variability	Low mean/low variability	Low mean/high variability	
	*N* = 1,397,024	*N* = 1,396,885	*N* = 1,396,820	*N* = 1,397,025	*p*-value
Age (years)	43.41 ± 11.88	44.14 ± 11.85	45.2 ± 12.05	47.46 ± 12.55	< .0001
Sex (male)	920,276 (65.87)	920,127 (65.87)	920,082 (65.87)	920,276 (65.87)	.9997
BMI (kg/m^2^)	23.21 ± 3.15	23.59 ± 3.18	23.96 ± 3.2	24.49 ± 3.22	< .0001
Waist circumference (cm)	78.96 ± 9.03	79.93 ± 9.05	80.94 ± 9	82.47 ± 8.88	< .0001
Systolic BP (mmHg)	120.58 ± 13.75	121.08 ± 13.78	121.66 ± 13.83	122.71 ± 13.96	< .0001
Diastolic BP (mmHg)	75.73 ± 9.57	76.06 ± 9.58	76.39 ± 9.58	76.89 ± 9.59	< .0001
Fasting glucose (mg/dL)	95.19 ± 18.58	95.96 ± 19.86	96.97 ± 21.37	99.11 ± 24.36	< .0001
Total cholesterol (mg/dL)	194.94 ± 29.91	193.9 ± 30.25	193.08 ± 30.64	191.19 ± 31.25	< .0001
Triglycerides (mg/dL)	96.05 (95.98–96.12)	105 (104.92–105.08)	115.27 (115.18–115.36)	135.24 (135.13–135.35)	< .0001
LDL-C mean (mg/dL)	111.66 ± 29.85	113.11 ± 29.99	114.01 ± 30.95	112.97 ± 31.82	< .0001
HDL-C mean (mg/dL)	61.93 ± 12.81	57.28 ± 11.16	53.55 ± 11.69	47.99 ± 10.87	< .0001
HDL-C SD (mg/dL)	3.68 ± 3.83	5.98 ± 5.62	8.03 ± 12.14	10.74 ± 13.1	< .0001
HDL-C CV (%)	5.6 ± 2.87	9.83 ± 3.83	13.66 ± 7.05	20.7 ± 10.65	< .0001
HDL-C VIM (%)	2.52 ± 0.95	5.04 ± 0.88	7.84 ± 1.26	14.92 ± 10.35	< .0001
HDL-C ARV (mg/dL)	4.51 ± 3.84	7.26 ± 6.47	9.56 ± 13.24	12.51 ± 13.09	< .0001
Current smoking	400,292 (28.65)	409,448 (29.31)	417,505 (29.89)	43,0470 (30.81)	< .0001
Alcohol use	121,692 (8.71)	116,184 (8.32)	109,071 (7.81)	97,937 (7.01)	< .0001
Regular physical activity	300,188 (21.49)	297,638 (21.31)	295,138 (21.13)	287,594 (20.59)	< .0001
Income (lower 25%)	218,360 (15.63)	231,608 (16.58)	252,314 (18.06)	286,618 (20.52)	< .0001
Diabetes mellitus	74,240 (5.31)	88,082 (6.31)	108,589 (7.77)	155,293 (11.12)	< .0001
Hypertension	250,289 (17.92)	275,699 (19.74)	309,843 (22.18)	383,983 (27.49)	< .0001
Any malignancy	21,057 (1.51)	22,118 (1.58)	24,426 (1.75)	30,085 (2.15)	< .0001

Data are expressed as mean ± SD, median (25–75%), or n (%). The low mean group includes participants in the first quartile of mean HDL-C (men: < 44.7 mg/dL, women: < 51.7 mg/dL), the high mean group includes those in the second to fourth quartiles, the low variability group includes the first to third quartiles of HDL-C variability, and the high variability group includes the fourth quartile (men: ≥ 10.4%, women: ≥ 8.5%).

*ARV* average real variability, *BMI* body mass index, *BP* blood pressure, *CV* coefficient of variation, *HDL-C* high-density lipoprotein cholesterol, *LDL-C* low-density lipoprotein cholesterol, *SD* standard deviation, *VIM* variability independent of the mean.

### Risk for Behçet’s disease according to HDL-C levels and variability

**Figure 1:**
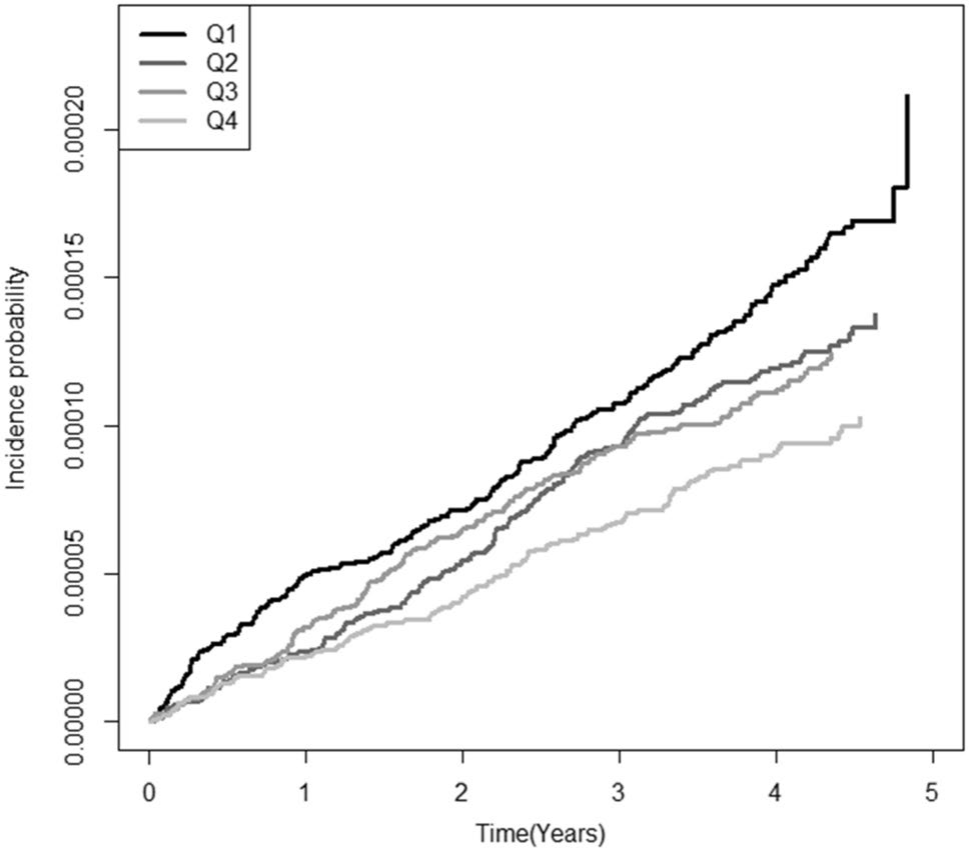
Kaplan–Meier curves showing the incidence of Behçet’s disease according to the quartiles (Q1–4) of high-density lipoprotein cholesterol levels.

**Figure 2:**
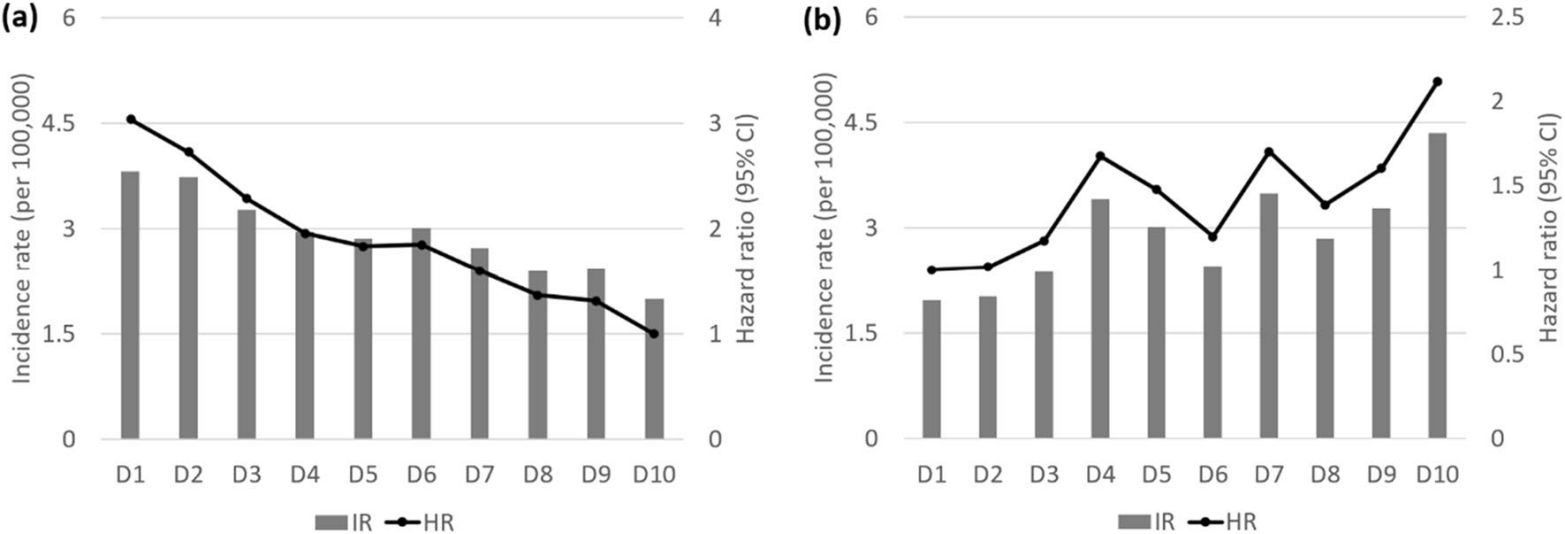
Incidence rates (IRs), hazard ratios (HRs), and 95% confidence intervals (CIs) of Behçet’s disease by deciles of high-density lipoprotein cholesterol mean (**a**) and variability (**b**). Adjusted for age, sex, body mass index, alcohol use, smoking, exercise, income, diabetes mellitus, hypertension, and mean high-density lipoprotein cholesterol.

There were 676 new cases of BD (0.01%) during the follow-up period. In the Kaplan–Meier survival analysis with BD diagnosis as the dependent variable, participants in the lowest quartile of HDL-C levels showed the lowest disease-free survival rate (Fig. 1). After adjusting for age, sex, BMI, alcohol use, smoking, exercise, income, DM, hypertension, and mean HDL-C levels, HRs for BD diagnosis were higher in decile (D) groups D1–6 of mean HDL-C compared to D10 (Fig. 2a) and in D4–10 of VIM compared to D1 (Fig. 2b). After adjusting for age, sex, alcohol use, smoking, exercise, income, BMI, DM, hypertension, and mean HDL-C levels, the HRs (95% CIs) for BD diagnosis were 1.335 (1.058–1.684) in the high-mean/high-variability group, 1.527 (1.211–1.925) in the low-mean/low-variability group, and 2.096 (1.670–2.630) in the low-mean/high-variability group compared to the high-mean/low-variability group (Table 2).

**Table 2:** Risk for Behçet’s disease according to HDL-C.

	Events (*n*)	Follow-up duration (person-years)	Incidence rate *	HRs (95% CIs)		
				Model 1	Model 2	Model 3
High mean/low variability	214	5,791,230.82	0.03695	1 (reference)	1 (reference)	1 (reference)
High mean/high variability	172	5,798,263.77	0.02966	1.248 (0.990–1.574)	1.334 (1.057–1.683)	1.335 (1.058–1.684)
Low mean/low variability	161	5,785,043.13	0.02783	1.350 (1.074–1.697)	1.526 (1.210–1.923)	1.527 (1.211–1.925)
Low mean/high variability	129	5,753,333.91	0.02242	1.719 (1.378–2.144)	2.094 (1.669–2.628)	2.096 (1.670–2.630)

Model 1: adjusted for age, sex, alcohol use, smoking, exercise, and income. Model 2: adjusted for model 1 plus body mass index, diabetes mellitus, and hypertension. Model 3: adjusted for model 2 plus mean HDL-C. The low mean group includes participants in the first quartile of mean HDL-C (men: < 44.7 mg/dL, women: < 51.7 mg/dL), the high mean group includes those in the second to fourth quartiles, the low variability group includes the first to third quartiles of HDL-C variability, and the high variability group includes the fourth quartile (men: ≥ 10.4%, women: ≥ 8.5%).

*CI* confidence interval, *HDL-C* high-density lipoprotein cholesterol, *HR* hazard ratio, *VIM* variability independent of the mean.

*Per 1000 person-years.

## Discussion

We found that a low mean level of HDL-C, and high variability, are associated with an increased risk for BD.

Although the pathophysiology of BD is not clear, infections in genetically predisposed individuals appear to play a role^4,16^. The genetic factors that increase the risk for BD include HLA-B*51 allele and single-nucleotide polymorphisms in interleukin 10, 23R, and 12RB2^4,12,16^. Triggers for BD include bacterial infections (such as *Streptococcus sanguis*, *Helicobacter pylori*, *and Mycoplasma*), viral infections (such as herpes simplex virus type 1, Epstein-Barr virus, hepatitis, and cytomegalovirus), and abnormal autoantigens (such as 60 kDa and 70 kDa heat-shock proteins, S-antigen, interphotoreceptor retinoid-binding protein, α-tropomyosin, and αβ-crystallin)^4,16^. Imbalances among natural killer cells, gamma delta T cells, and neutrophils may also be involved in the development of BD. Endothelial dysfunction and neutrophilic vascular inflammation cause thrombosis in BD patients. CD4 + T-helper cell subtypes 1, 2, 17, and 22, regulatory T cells, and cytokines involved in the adaptive immune system are also important in the pathogenesis of BD^16^.

Decreased levels of protective HDL-C reduce the activity and expression of endothelial nitric oxide synthase, leading to oxidative stress and damage to the vascular endothelium^17^. Recent studies have suggested that HDL-C protects the endothelium by stimulating the production of endothelial anti-atherogenic nitric oxide. HDL-C promotes endothelial repair and also has antioxidant, anti-inflammatory, and antithrombotic effects^18–20^. HDL-C infusion inhibits neutrophil activation in patients with peripheral vascular disease^21^ and reduces pro-inflammatory cytokine (interleukin-6, chemokine ligand 2, and tumor necrosis factor α) production in mice^22,23^. In a recent study, statins reduced T cell immunoglobulin- and mucin-domain-containing molecule-3 expression on natural killer and natural killer T cells, associated with an elevated HDL-C/total cholesterol ratio and decreased total cholesterol and LDL levels^24^. Therefore, reduced HDL levels induce endothelial dysfunction and activate neutrophils and natural killer cells, suggesting that HDL is involved in the pathogenesis of BD.

Clustering of triglyceride, HDL-C, glucose, waist circumference, and blood pressure was seen in a pattern that emulates metabolic syndrome. Among them, low HDL-C has been found to be related to MI and stroke in patients with metabolic syndrome^25^. In addition, high HDL-C variability is associated with an increased risk for MI, stroke, end-stage renal disease, and diabetes^7–11,26–28^. It may cause plaque instability due to impaired cholesterol efflux from peripheral tissues and macrophages^26,29^. The result of our study suggest that HDL-C variability increases the risk for BD, similar to other diseases described above.

Our study had some limitations. Although we used a 5-year washout period before the index year, reverse causation cannot be ruled out because this was a retrospective study. There are no data on the effects of duration of HDL-C abnormalities on the risk for BD, and there was a short follow-up duration in our study. In addition, exclusion of participants who had less than three medical examinations may have introduced selection bias in our study. Because most patients with dyslipidemia are taking statins, the effect of statins on cholesterol variability should be considered. However, since statins have little long-term effect on HDL-C, it is considered that the effect on variability of HDL-C is also small^7,8^.

Despite these limitations, our study had several strengths. For one, we used data from a large nationwide database representative of the entire Korean population to determine the relationship between HDL-C variability and BD. To the best of our knowledge, this is the first study on the association between HDL-C levels and risk for BD. Low mean levels of HDL-C, and high variability, were independent predictors of BD. Physicians should be aware of the increased risk for BD in such patients. Treatment of BD should also focus on normalizing the HDL-C levels. Further studies on the effects of HDL-C variability on endothelial dysfunction are required to evaluate the mechanisms underlying the relationship between HDL-C and BD.

In conclusion, in order to lower the risk of BD, it is important to raise HDL-C (more than 58.7 mg/dL in men and more than 67.5 mg/dL in women) and keep this condition constant for lowering variability. Quitting smoking, oral estrogen replacement therapy, extensive aerobic exercise, and treatment with niacin, statins, or fibrates may help increase HDL-C^30–32^.

## Methods

### Study design and database

The Korean National Health Insurance System (NHIS) uses billing records of healthcare providers to collect data on age, sex, demographic variables, treatment records, general health examinations, lifestyle, and behavior of patients. Healthcare providers are advised to perform standardized medical examinations every 1 or 2 years. Enrollment in the NHIS is compulsory for all of the > 50 million Korean residents^33,34^.

### Study population

**Figure 3:**
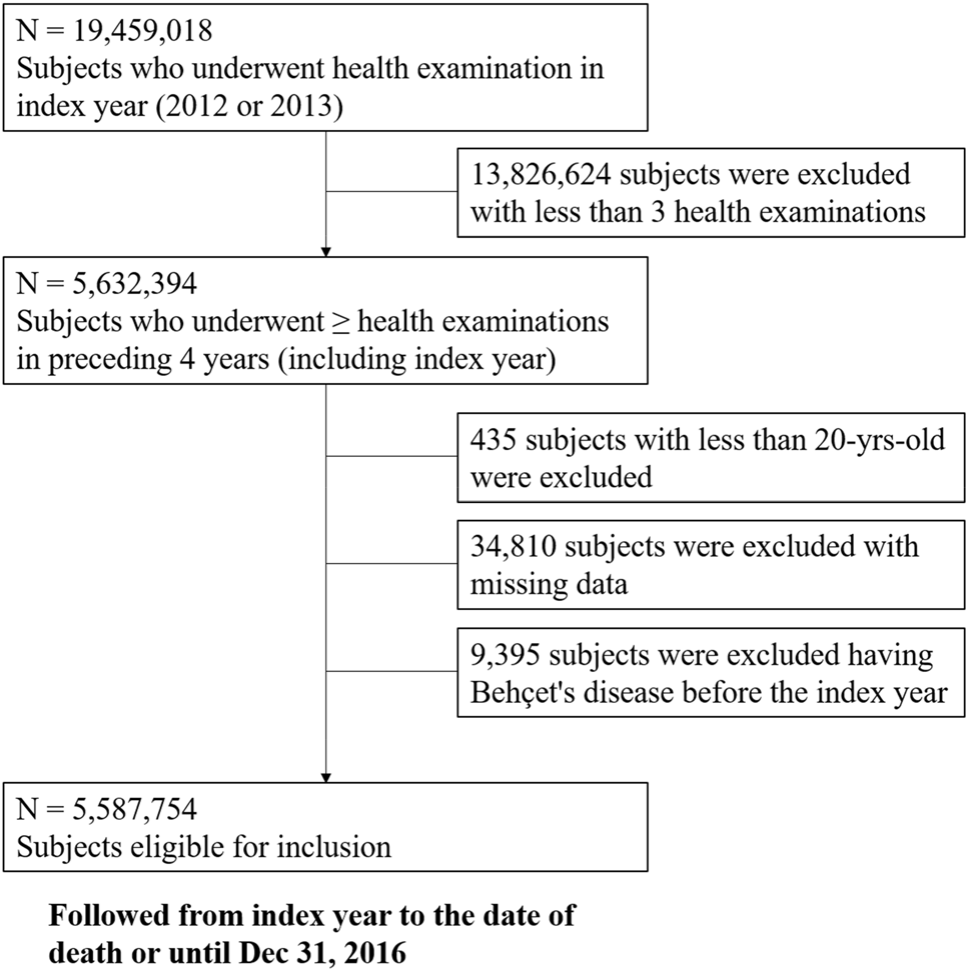
Flowchart of the selection of study participants.

We collected data from 19,459,018 participants in the NHIS who underwent medical checkups between 2012 and 2013 (index year), and had been enrolled in NHIS for at least 5 years before the checkup (i.e., washout period: 2007–2011). We selected 5,632,394 participants who underwent a medical checkup in the index year and ≥ 2 checkups during the preceding 3 years. After exclusion of participants of age < 20 years (*n* = 435) or missing data (*n* = 34,810), data from 5,587,754 participants was included in this study (Fig. 3). Ethical approval was obtained from the Institutional Review Board of Uijeongbu St. Mary’s Hospital, Catholic University of Korea (UC18ZESI0094). Informed consent was not required as the data used for this study were anonymized. All methods were carried out in accordance with relevant guidelines and regulations. The need of the written informed consent has waived by the ethics committee of institutional review board of the Korean National Institute for Bioethics Policy and the Catholic University of Korea Institutional Review Board.

### Measurements and definitions

Body mass index (BMI) was calculated by dividing the weight (kg) by the square of height (m^2^), and obesity was defined as BMI ≥ 25 kg/m^2^^35^. “Regular physical activity” was defined as ≥ 20 min of vigorous physical activity ≥ 3 times/week or ≥ 30 min of moderate-intensity physical activity ≥ 5 times/week.

Household income was dichotomized at 25% of monthly income^36^. Diabetes mellitus (DM) was recorded as being present if the fasting blood glucose level was ≥ 126 mg/dL or there was ≥ 1 claim/year for International Classification of Disease, tenth revision (ICD-10) codes E10–14 and ≥ 1 claim/year for antidiabetic drug prescriptions. Hypertension was recorded as being present if the blood pressure was ≥ 140/90 mm Hg or there was ≥ 1 claim/year for ICD-10 codes I10–15 and ≥ 1 claim/year for antihypertensive drug prescriptions. Dyslipidemia was recorded as being present if the total cholesterol was ≥ 240 mg/dL or there was ≥ 1 claim/year for ICD-10 code E78 and ≥ 1 claim/year for lipid-lowering drug prescriptions. Self-administered questionnaires were used to document social behaviors, including smoking, alcohol use, and physical activity.

### Definition of HDL-C variability

HDL-C variability was calculated using HDL-C levels measured at two different health screenings. HDL-C variability was measured with the coefficient of variation (CV), variability independent of the mean (VIM), and average real variability (ARV). CV was calculated as (standard deviation [SD]/mean) × 100. VIM was calculated as (SD/mean^β^) × 100, where β was the regression coefficient^37,38^. ARV was the average absolute difference between consecutive HDL-C levels^39^.

### Definition of low mean HDL-C and high HDL-C variability

Mean HDL-C values differ between men and women; therefore, we used sex-specific cutoff values (Table 3). Participants with HDL-C levels in the lowest quartile (quartile 1) were included in the low-mean HDL-C group, and those with HDL-C levels in the remaining three quartiles (quartiles 2–4) were included in the high-mean HDL-C group. Participants with HDL-C variability in the highest quartile (quartile 4) were included in the high-variability group, and those with HDL-C variability in the remaining three quartiles (quartiles 1–3) were included in the low-variability group.

**Table 3:** Cut-off values for mean high-density lipoprotein cholesterol levels in men and women.

	1st quartile	2nd quartile	3rd quartile
**Men**			
Mean (mg/dL)	44.7	51	58.7
SD (mg/dL)	3.6	5.5	8.2
CV (%)	7.2	10.8	15.6
VIM (%)	4.2	6.7	10.4
ARV (mg/dL)	4	6.5	10
**Women**			
Mean (mg/dL)	51.7	59.3	67.5
SD (mg/dL)	4.2	6.6	9.6
CV (%)	7.3	11.2	16.2
VIM (%)	3.3	5.4	8.5
ARV (mg/dL)	5	7.7	11.7

*ARV* average real variability, *CV* coefficient of variation, *SD* standard deviation, *VIM* variability independent of the mean.

### Study outcomes and follow-up

The end point of this study was a diagnosis of BD, defined as documentation of ICD-10 codes M35.2 or V139 (rare intractable diseases). The study participants were followed up from baseline to BD diagnosis or until December 31, 2016, whichever came earlier. The median follow-up duration was 4.22 (4.01–4.55) years.

### Statistical analysis

Baseline demographics are presented as mean ± SD, median (interquartile range 25–75%), or *n* (%). Participants were grouped using HDL-C mean and CV quartiles. Hazard ratios (HRs) and 95% confidence interval (CI) values were calculated using the Cox proportional-hazards model. The HRs (95% CIs) of the low-mean HDL-C and high-variability groups were compared to those of the high-mean HDL-C and low-variability groups, respectively. Kaplan–Meier estimates were used to calculate the cumulative HRs for quartiles of HDL-C mean and variability as well as groups of combinations of mean and variability. These HRs were used in the Schoenfeld residuals test to evaluate the proportional hazards function. There was no significant departure from proportionality of hazards over time. A proportional-hazards model was applied after adjusting for age, sex, BMI, alcohol use, smoking, exercise, income, DM, and hypertension. We used stratified analysis and a likelihood-ratio test to evaluate potential modifier effects of age, sex, obesity, DM, hypertension, malignancy, and use of lipid-lowering agents. Statistical analyses were performed using SAS software (version 9.4; SAS Institute Inc., Cary, NC, USA), and *p*-values ≤ 0.05 were considered to indicate significance.

## Data Availability

The data that support the findings of this study are available within the article.
